# Physiological load and breath-holding in artistic swimming: a scoping review establishing historical baselines and evidence gaps in the context of the 2022–2025 rule changes

**DOI:** 10.3389/fphys.2026.1855762

**Published:** 2026-06-18

**Authors:** QiTing Zhou, Yunan Xiang, Soi Po Wong, U. Kei Wong, Yirui Zhao, Wenjing Fan

**Affiliations:** 1 Faculty of Education, University of Macau, Macao, Macao SAR, China; 2 School of Sports Training, Wuhan Sports University, Wuhan, China

**Keywords:** apnea, artistic swimming, physiological load, rule changes, scoping review

## Abstract

**Background:**

The 2022–2025 scoring system assigns higher difficulty to complex hybrid elements and acrobatics, which consequently necessitate prolonged underwater immersion. Therefore, it is hypothesized that maximizing scores through extended underwater exertion potentially exposes artistic swimmers to increased physiological demands. Since most quantitative data predates the 2022 rules, traditional routine evidence may underrepresent current competitive stress.

**Objective:**

This scoping review systematically map literature on artistic swimmers’ physiological load and apnea characteristics, establishing a historical baseline to identify critical evidence gaps introduced by the 2022–2025 regulatory changes.

**Methods:**

Following PRISMA-ScR guidelines and the PCC framework, a systematic search across four major databases identified peer-reviewed original research reporting physiological load and/or apnea-related outcomes in competitive artistic swimmers.

**Results:**

Synthesis of 36 articles revealed: (1) Routine execution triggers autonomic conflict (concurrent sympathetic/parasympathetic activation), producing broad heart rate ranges (~56.5–203.8 bpm) Additionally, chronic sport-specific training is linked to distinct cardiovascular adaptations. (2) Apnea duration in late traditional (pre-2022) routines constitutes 59%–64% of total performance time. Post-routine blood lactate concentrations are reported ~5.93–11.5 mmol/L. The revised regulations assign higher scoring weights to complex hybrid elements(officially defined as a combination of five or more lower-limb movements performed with intentional apnea),which aligns with increased underwater duration (Time Underwater, TU), higher anaerobic metabolic contributions, and instances of post-exercise hypoxemia. (3) The “Base Mark” penalty mechanism (the minimum degree of difficulty applied for non-conforming or failed elements) correlates with increased cognitive load. Studies also report stress-induced cortisol elevations and altered recovery of heart rate variability under these conditions. (4) Adolescent athletes exhibit still-developing anaerobic and cardiovascular systems. Consequently, based on legacy baselines, recent discussions hypothesize that younger athletes performing the high-frequency, prolonged underwater choreography promoted by the new scoring system may be exposed to increased acute hypoxic stress.

**Conclusions:**

Artistic swimming’s exertion and prolonged apnea involve autonomic co-activation, high anaerobic demands, and specific psychophysiological responses. Based on theoretical extrapolation from established physiological baselines, it is hypothesized that the 2022–2025 scoring system increases athletes’ exposure to cardiovascular and metabolic loading, presenting safety considerations for adolescents. Since most evidence predates 2022, longitudinal research under valid competitive conditions is necessary to assess the new regulations’ physiological impacts.

**Systematic Review Registration:**

https://osf.io/dxapc/, identifier 10.17605/OSF.IO/DXAPC.

## 1 Introduction

Artistic swimming requires athletes to execute highly choreographed technical movements while experiencing high physiological loads (Viana et al., 2019). The sport’s kinematic characteristics, including surface jumps and explosive limb actions, increase sympathetic stimulation to the heart and elevate heart rate (Rodríguez-Zamora et al., 2012). Concurrently, facial immersion and apnea (breath-holding) trigger the diving reflex. This response induces parasympathetically driven bradycardia (Konstantinidou and Soultanakis, 2016). The simultaneous activation of both autonomic branches, known as autonomic co-activation, may alter myocardial electrical heterogeneity and repolarization. These factors have been linked to potential arrhythmogenesis in other contexts (Shattock and Tipton, 2012). In a recent clinical commentary, Williams et al. (2024) hypothesized that specific autonomic interaction creates a complex physiological environment, making cardiovascular responses highly variable and difficult to predict.

Early empirical research focused on the acute physiological responses to independent compulsory Figure events and complete routines. These investigations monitored heart rate fluctuations, decreases in alveolar oxygen partial pressure (to approximately 60 mmHg), and blood lactate concentrations. This early work provided an initial foundation for quantifying the sport’s internal load (Figura et al., 1993). Under traditional choreographic rules, elite athletes spent half or more of their total routine time in a state of facial immersion and apnea (Rodríguez-Zamora et al., 2014a),with the overall technical complexity heavily dependent on the structural density of these apneic connective movements (Shul’ga and Rudkovskaya, 2013). Despite prolonged apnea and underwater exertion, core physiological indicators like peak heart rate, blood gas metabolism, and blood lactate remain quantifiable using conventional monitoring techniques (Bentley et al., 2022). Over time, research in artistic swimming has shifted from relying on isolated training metrics to assessing physiological profiles in real-world competitive settings. This methodological progression helps address earlier knowledge gaps and provides observational data for evaluating the specific physiological demands inherent to the sport (Rodríguez-Zamora et al., 2012).

For the 2022–2025 Olympic cycle, World Aquatics introduced a revised evaluation system, which has been continuously updated through its latest technical manuals (World Aquatics, 2022, 2025a)This update establishes stringent assessment criteria for the base marks of acrobatics and hybrids, hybrid family bonuses, and required technical elements. These regulatory changes tend to assign higher difficulty values to complex technical elements and acrobatics, making prolonged underwater immersion a downstream consequence rather than a directly rewarded criterion (Elsherif, 2025; Williams et al., 2024). The drive to maximize scores through the accumulation of complex hybrid elements and acrobatic sequences—which inherently require prolonged breath-holding as a downstream consequence—may impose unique physiological demands on athletes. In the context of high training volumes, it is theorized that frequent and prolonged apnea could be an additional stressor related to Relative Energy Deficiency in Sport (RED-S) and potential long-term systemic health concerns, though direct causal evidence remains limited (Elsherif, 2025). Additionally, the accompanying high-intensity technical movements contribute to rapid blood lactate accumulation. These levels have been reported to show moderate accumulation (5.93 ± 1.41 mmol/L) compared to earlier records (Iglesias et al., 2025). The superimposition of vigorous exertion and extended apnea can induce autonomic conflict. Recent perspective papers suggest this interaction may expose athletes to elevated hypoxic stress, with extreme cases in aquatic environments historically linked to shallow water blackout or syncope (Williams et al., 2024). In response to these concerns, European Aquatics (EA) has implemented further regulations regarding category-specific underwater durations (specifically targeting the Junior category) (SwimSwam, 2025).

Although previous research has investigated the physiological characteristics of artistic swimming (Viana et al., 2019), the sport’s technical rules have undergone frequent evolution (Mountjoy et al., 2010; Nakashima et al., 2013). This evolution is compounded by notable inconsistencies in measurement indices and quantification methods across historical studies (Bante et al., 2007; Figura et al., 1993; Poole et al., 1980; Rodríguez-Zamora et al., 2012; Yamamura et al., 1999). The vast majority of quantitative data regarding physiological load was collected prior to the 2022 rule implementation. This chronological gap suggests that historical data derived from traditional routines may not accurately characterize the physiological stress currently experienced by elite athletes. Given the combination of high-density movement sequences and prolonged apnea, there remains a lack of systematic data integration and evidence mapping regarding the physiological loads encountered in authentic competitive environments under the new scoring system (Viana et al., 2019).

Physiological research in artistic swimming continues to develop, yet the available evidence remains fragmented. A scoping review methodology provides a transparent and rigorous approach to map this literature (Munn et al., 2018). Therefore, this scoping review aims to systematically synthesize the existing literature to establish a comprehensive baseline of physiological and apneic demands in artistic swimming. By mapping this legacy data, we aim to identify critical evidence gaps and theoretically extrapolate the potential physiological impacts of the 2022–2025 rule changes. Specifically, this review addresses the following four primary research questions:

RQ1: What is the existing evidence regarding the acute physiological load and breath-holding characteristics in elite artistic swimming athletes in the context of the World Aquatics, 2022–2025 scoring system?

RQ2: What is the existing evidence mapping the reliance on anaerobic energy contribution and the characteristics of post-exercise hypoxemia across different regulatory periods?

RQ3: What is the existing evidence concerning the psycho-physiological interactions, specifically regarding heart rate variability (HRV) and cortisol responses, associated with the “Base Mark” assessment environment?

RQ4: How does the current literature map the physiological adaptation patterns across different age groups and event types?

## 2 Methods

### 2.1 Review design

This study was designed as a scoping review to map the existing literature on physiological load and breath-holding in artistic swimming. The review was conducted in accordance with the Preferred Reporting Items for Systematic Reviews and Meta-Analyses extension for Scoping Reviews (PRISMA-ScR) guidelines. The protocol for this scoping review was registered on the Open Science Framework (OSF) on January 31, 2026 (DOI: 10.17605/OSF.IO/DXAPC). Although formal registration occurred after the execution of the database search (January 12–19, 2026), it was completed strictly prior to the commencement of data extraction, full-text thematic mapping, and qualitative synthesis. This sequencing ensured that the analytical framework and PCC criteria were established beforehand, effectively mitigating outcome reporting bias. A scoping review methodology was chosen due to the heterogeneity of study designs, outcome measures, and research contexts in this field, as well as the exploratory nature of the research questions.

### 2.2 Eligibility criteria (PCC framework)

The eligibility criteria for this scoping review were developed using the Population, Concept, and Context (PCC) framework, as summarized in **Table 1**.

**Table 1:** PCC framework.

Component	Inclusion criteria	Exclusion criteria
Population	Competitive artistic swimmers, across explicitly defined age categories (e.g., U12, Youth, Junior, Senior) sexes, and competitive levels.	Individuals participating exclusively in rehabilitative or non-competitive swimming; athletes from non-artistic swimming disciplines.
Concept	Studies investigating specific physiological domains (cardiovascular, metabolic, respiratory, psychophysiological)and apnea-related physiological characteristics during defined routine types (e.g., solo, duet, team; technical, free, acrobatic); research addressing the impact of the 2022–2025 new scoring system.	Articles lacking physiological monitoring or solely investigating psychological or biomechanical factors without physiological interactions; studies exclusively examining routines under previous rule frameworks with no baseline reference value.
Context	Specific testing environments including laboratory tests, simulated routines in pools, and in-competition monitoring within competitive sporting events or systematic training environments.	Non-systematic recreational activities; environments lacking professional coaching or experimental monitoring.
Evidence Type	Peer-reviewed empirical studies, case reports, systematic reviews, and grey literature (e.g., official manuals).	Book reviews, editorials, non-academic reports, or abstracts without accessible full texts.

### 2.3 Search strategy

A systematic electronic search of PubMed, Web of Science Core Collection, and Scopus was conducted up to January 19, 2026. Google Scholar was utilized as a supplementary tool (restricted to 2021–2026 literature to specifically capture recent grey literature and emerging evidence directly leading up to and following the 2022 regulatory changes). The search syntax combined concepts of artistic swimming, physiological load, and breath-holding using Boolean operators (“AND”, “OR”). Only peer-reviewed articles published in English were considered, and the reference lists of included studies were hand-searched to identify additional relevant records. The complete, reproducible search strategy for all databases is provided in **Supplementary Material S1**.

### 2.4 Study selection and data extraction

Following the removal of duplicates, two independent reviewers screened titles and abstracts, which was subsequently followed by a full-text evaluation against the predefined eligibility criteria. Any discrepancies were resolved via discussion or by consulting a third reviewer. Data extraction was performed independently by two reviewers using a pilot-tested standardized form. The extracted data items included: (1) publication and participant characteristics, (2) study design and setting, (3) physiological and breath-holding parameters, (4) measurement tools, and (5) key findings relevant to the research questions.

### 2.5 Synthesis of results

Due to the substantial heterogeneity in study designs, outcome measures, and contexts, a meta-analysis was not feasible. Instead, the extracted data were synthesized descriptively through thematic mapping. The findings were presented in both narrative and tabular formats to address the research questions and identify current knowledge gaps in the literature.

## 3 Results

### 3.1 Selection and characteristics of sources of evidence

**Figure 1:**
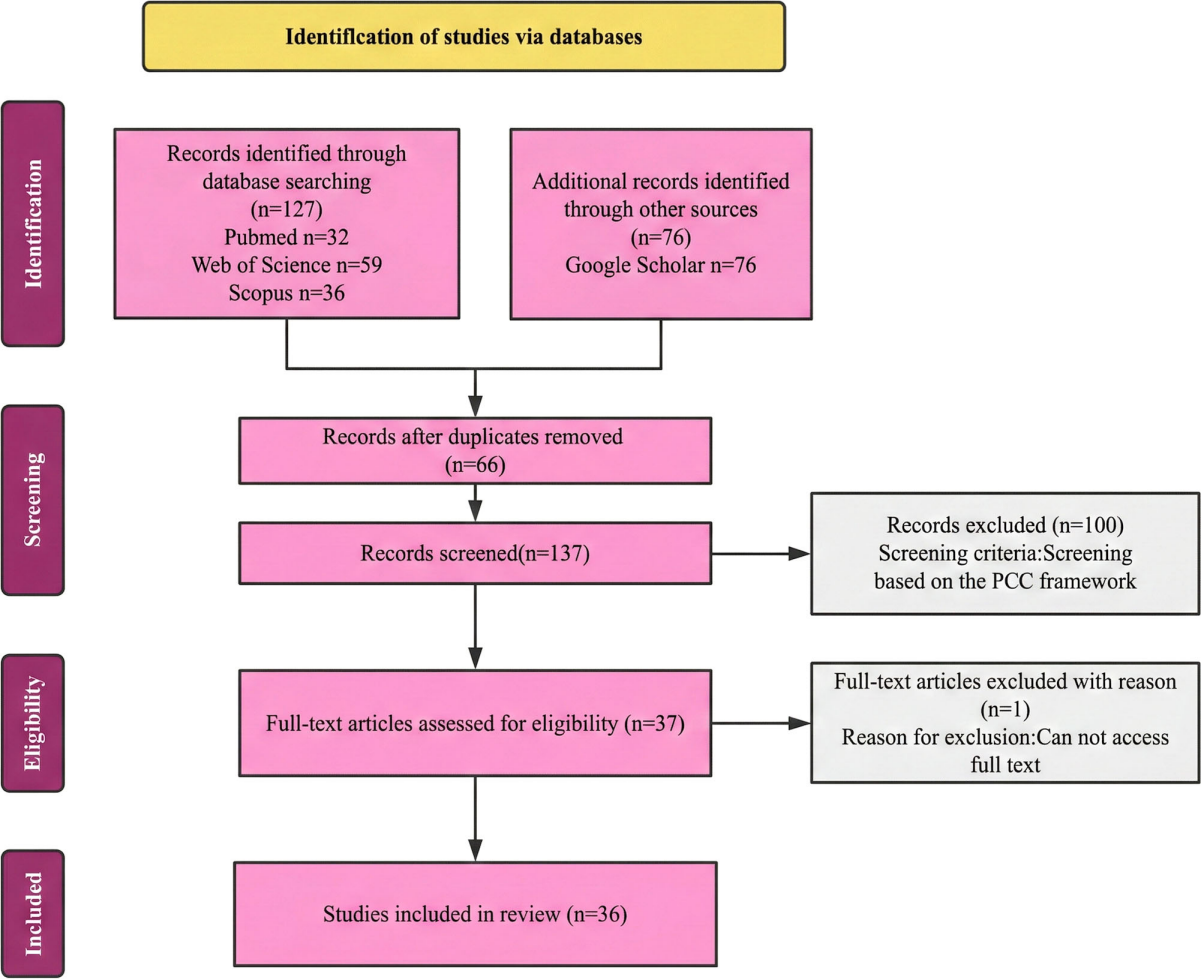
PRISMA-ScR flow figure.

In accordance with the pre-registered PRISMA-ScR guidelines, the initial literature search identified a total of 203 records across four electronic databases (PubMed, n = 32; Web of Science, n = 59; Scopus, n = 36; additional records identified via supplementary searches in Google Scholar, n = 76). Following the removal of 66 duplicates, the titles and abstracts of the remaining 137 records were screened based on the predefined Population-Concept-Context (PCC) framework, resulting in the exclusion of 100 ineligible records. Subsequently, the remaining 37 articles underwent full-text evaluation for eligibility. During this phase, one article was excluded because the full text could not be retrieved. Ultimately, 36 original studies met all eligibility criteria and were included in the final qualitative synthesis (**Figure 1**). The detailed characteristics of all included studies are summarized in **Table 2**.

**Table 2:** Summary of study characteristics, physiological responses, and contextual background in artistic swimming.

Study & participants	Routine type & rules	Standardized physiological & apnea outcomes	Key findings	Methodological caveats
Rodríguez-Zamora et al. (2018); Observational field study; n=34 (Elite)	Solo, Duet, Team;Old Rules	Apnea Data: Duration: Solo 64%, Duet 59%.	Artistic swimming routines induce moderate net lactate accumulation compared to freediving, likely due to intermittent breathing facilitating partial lactate oxidation.	Limited group sizes; Sampling differences
Jones and Cooper (2018); Observational study; n=6 (Elite)	Duet; Old Rules	NIRS: Rapid decline in vastus lateralis TSI% with stable prefrontal cortex TSI%.	Despite extreme breath-holding and vigorous exercise, elite artistic swimmers maintain stable cerebral oxygenation, indicating a protective adaptation akin to the dive reflex.	Small sample size (n=6); Simulated routines
Rodríguez-Zamora et al. (2014a); Observational comparative study; n=10 (Elite)	Technical Duet, Free Duet; Old Rules	HR Data:Peak: 184.8 ± 6.6 bpm,Min:50.8 b·min−1(Subject 5);Psychophysiological Data: RPE:7.5 - 7.9.	Internal physiological load and RPE are highly correlated with the frequency and duration of bradycardia events induced by apneic episodes during routines.	Small sample size (n=10); Simulated and in-competition
García et al. (2021); Observational repeated-measures study; n=11 (Elite)	Apneic swimming, figures and choreography;Old Rules	Respiratory Data: Decreased DLCO.	Elite artistic swimmers exhibit superior lung volumes and diffusing capacities, which dynamically adapt to breath-holding and intense training without clinical pulmonary impairment.	Small sample size (n=11); Simulated training segments
Alentejano et al. (2008); Time-motion observational study; n=11 (Elite)	Solo; Old Rules	Apnea Data:Duration:59%.	Routine apnea durations stay within physiologically safe limits (<40s) and show no significant correlation with technical merit scores in elite solo performances.	Video analysis only; No physiological data
Bante et al. (2007); Observational comparative study; n=16 (Senior & Junior National)	Simulated routine (figures);Old Rules	Lactate Data: Post-routine levels were similar between junior and senior athletes.	Senior and youth athletes present similar cardiorespiratory responses, though seniors exhibit higher post-maximal test lactate levels indicating greater anaerobic maturity.	Simulated routine in training; Small sample size per group
Badau (2024); Interventional cross-sectional study; n=92 (Junior/Beginner, Ages 7-14)	Apnea tests (static);New Rules (2022-25)	Apnea Data: Baseline apnea tolerance is limited by somatic maturity and biological age	Age significantly dictates breath-holding capacity in 7-8-year-old beginners, after which specific training experience becomes the predominant determinant.	Static apnea tests only; No full routine
Ntomali et al. (2025); Randomized crossover experimental study; n=12 (Elite/National)	Free Team; Standard/FINA Guidelines	Lactate Data: Often lower than baseline conditions.	Mental fatigue significantly impairs technical performance, jump height, and glycolytic activation in artistic swimming without affecting maximal sprint capability.	Simulated routine in training; Mental fatigue intervention
Dzimbova and Markov (2023); Longitudinal observational study; n=9 (Junior)	Wingate and Sargent tests;Old Rules	Power: Wingate peak power improved from 147.12 to 210.17 W.	One year of specialized artistic swimming training significantly enhances anaerobic capacity, explosive power, and lean muscle mass in junior athletes.	Small sample size (n=9); Lab tests only
Naranjo et al. (2006); Experimental comparative study; n=25 (13 Elite AS, 12 Controls), (High competitive level)	Free routine + cycle ergometer;Old Rules	Apnea Data:Duration: 45.11%.	Elite artistic swimmers demonstrate specific respiratory adaptations, enabling efficient CO2 elimination during intermittent breath-holding exercise without excessive lactate elevation.	Simulated routine on cycle ergometer; Lacks ecological validity
Alentejano et al. (2010); Experimental comparative study; n=30 (15 AS, 15 Controls),(Elite)	Breath holding (BH) tests;Old Rules	Not specifically detailed; cited for methodological context (inclusion of untrained control groups).	Artistic swimmers exhibit significantly longer breath-holding times and more pronounced diving bradycardia, likely due to diminished psychological fear and superior physiological oxygen conservation.	Simulated breath-holding test; No full routine
Davies et al. (1995); Observational field study; n=9 (Elite)	Set figures and Free program; Old Rules	Respiratory Data: Acute symptoms of severe hypoxia (cyanosis and transient cognitive impairment).	Prolonged underwater sequences under old regulations induce dangerous levels of hypoxia, cyanosis, and mild confusion, underscoring the necessity of limiting apneas.	Small sample size (n=9); Isolated figures only
Coates et al. (2022b) (Cardiac Remodeling); Cross-sectional study; n=90 total, 23 AS (Elite)	Not specified; Old Rules	Cardiac: Increased left ventricular posterior wall thickness and left atrial area.	Elite artistic swimmers undergo unique cardiac structural remodeling, characterized by disproportionate left ventricular wall thickening driven by repetitive apneic pressure loads.	Resting assessment; No routine performed
Chatard et al. (1999); Interventional study; n=13 (Junior/Highly trained)	Team routine; Old Rules	Metabolic Data: Anaerobic demands comparable to a 400-meter maximal all-out swim.	A 5-week technical training block improves routine scores but decreases maximal aerobic power, revealing that technical scores depend more on skill execution than physiological enhancement.	Simulated routine in training
Coates et al. (2022a) (LV Function); Cross-sectional study; n=90 total, 23 AS (Elite)	Isometric handgrip;Old Rules	HR Data:Min/Rest:57 ± 9 bpm.	Elite artistic swimmers develop specific left ventricular functional adaptations to counteract the low-volume, high-pressure hemodynamic challenges of apneic exercise.	Lab test only; No swimming routine
Figura et al. (1993); Observational/Experimental study; n=6 (National)	Compulsory figures and Freestyle;Old Rules	Respiratory Data: pAO2 dropped to ~60 mmHg;Autonomic Data: Heart rate fluctuations (bradycardia/tachycardia).	Diving bradycardia induced by breath-holding powerfully overrides exercise-induced tachycardia, while extreme apneas lead to physiological breaking points via oxygen depletion.	Small sample size (n=6); Simulated routines
Rodríguez-Zamora et al. (2012); Observational field study; n=34 (Elite)	Solo, Duet, Team (Technical & Free);Old Rules	HR Data:Peak: 191.3 ± 9.2 bpm (Junior) and 192.4 ± 8.0 bpm (Senior).	Anticipatory pre-activation and pronounced diving bradycardia are critical predictors of competition scores, highlighting the adaptive metabolic response of moderate lactate accumulation.	Missing data due to HR recording failures
Fan et al. (2024); Randomized controlled trial; n=14 (Elite/National)	50 m diving, 25 m torpedo;New Rules (2022-25)	Not specifically detailed; cited as an example of multi-week aquatic interventions under the new 2022–2025 rules.	High-intensity inspiratory muscle resistance combined with strength training significantly enhances aerobic capacity and underwater breath-holding performance.	Specific aquatic tests; No full routines
Solana-Tramunt et al. (2019); Observational study; n=12 (Elite)	Technical Team;Old Rules	HR Data: Autonomic fluctuations (apnea-induced diving reflex and sympathetic excitation).	Assessing autonomic recovery via HRV is heavily confounded by the intense parasympathetic activation (diving bradycardia) induced by frequent apneas.	Simulated routine in training; Missing HR data
Podrihalo et al. (2021); Observational predictive study; n=30 (Junior)	Functional tests;Old Rules	Respiratory Data: Hypoxic tolerance (as a pivotal factor).	Superior breath-holding capacity and specific morpho-functional indicators serve as highly reliable predictors for talent identification and competitive success.	Lab tests only; Non-elite sample
Guézennec et al. (2026); Quasi-experimental study; n=15 (Regional/Junior)	275-m underwater swim test (UWST) & Lab tests;General	Respiratory Data: Arterial oxygen saturation (SpO2) falling to 88.4%	Combining end-expiratory breath-hold sprints with inspiratory muscle training augments peak oxygen uptake and muscle deoxygenation capacity.	Small sample size (n=15); Specific aquatic UWST instead of a real AS routine; Quasi-experimental design
Buscà et al. (2020); Randomized crossover study; n=12 (Elite)	Technical Team;Old Rules	Endocrine Data: Elevated stimulated salivary cortisol (SC) Autonomic Data: HRV (LnRMSSD) unaffected.	Wearing a jaw-repositioning intra-oral device elevates salivary cortisol without improving metabolic recovery, acting as an unadvisable additional stressor.	Simulated routine in training with intra-oral device
Alentejano et al. (2012); Experimental comparative study; n=30 (Competitive vs Control)	Underwater arm cranking;Old Rules	HR Data:Robust bradycardia during 20s/25s apneas; Apnea Data:Max single bout: 25s.	Trained artistic swimmers display a blunted ventilatory response and robust bradycardia during underwater exercise, demonstrating superior physiological adaptations to hypoxia.	Simulated lab test; No routine
Iglesias et al. (2025); Quasi-experimental repeated-measures study; n=16 (Elite)	Free Duet;Old Rules	HR Data: Peak 203.8 ± 5.0 bpm; Min 71.9 ± 16.6 bpm;Lactate Data: Post-routine 5.93 ± 1.41 mmol/L.	Extreme metabolic load in duet routines stems from the cumulative effect of high-intensity exercise and repeated apneas rather than isolated short breath-holding events.	Segmented routine protocol; VO2 estimated via retro-extrapolation
Elsherif (2025); Observational video analysis; n=9 teams (Elite/Olympic)	Free routine;Old Rules	Apnea Data: Duration: 57.8% (Old Rules data used for New Rules TU3 theoretical modeling).	Extending breath-holding times beyond physiological limits reduces performance quality; an optimal 1:1 work-to-breathing ratio best balances technical scores and metabolic sustainability.	Video analysis only; No physiological data
Baker et al. (2024); Cross-sectional study; n=36 (Elite/Varsity/Control)	Isometric handgrip;Old Rules	Cardiac: aortic elastic properties.	Elite artistic swimmers exhibit distinct cardiac structural characteristics and preserved diastolic function to counteract extreme hydrostatic and apneic pressure loads.	Resting and handgrip test only; No routine
Sprenger et al. (2025); Randomized double-blind crossover study; n=17 (Elite adolescent)	Simulated duet routine;General	Lactate Data:Post-routine:8.4 ± 0.9 mmol/L (Baseline/Placebo),9.3 ± 1.0 mmol/L (Using NaHCO_3)_	Individualized sodium bicarbonate supplementation effectively mitigates perceived exertion and selectively enhances propulsion scores by buffering extreme metabolic acidosis.	Small sample size for intervention (n=7); Simulated routine
Rodríguez-Zamora et al. (2014b); Observational field study; n=17 (Elite)	Solo, Duet (Technical & Free);Old Rules	HR Data: Heart rate extremes (cited as context for old rules).	Perceived exertion is strongly correlated with breath-holding duration, lactic acidosis, and the severity of diving bradycardia.	Missing data due to HR recording failures
Costa et al. (2018); Observational study; n=21 (Collegiate)	Not specified;Old Rules	Metabolic: Low energy availability (EA)	Collegiate artistic swimmers frequently suffer from severely low energy availability, predisposing them to bone health complications and the female athlete triad.	Resting measurements only; No routine
Rančić et al. (2024); Cross-sectional study; n=60 (Junior)	Not specified; General	HR Data:Min/Rest: 72.7 ± 9.18bpm.	Rhythmic breathing and breath-holding demands in aquatic sports confer significantly superior lung function in artistic swimmers compared to terrestrial aesthetic athletes.	Resting measurements only; Lab tests;No AS routine tested
Schaal et al. (2013); Randomized crossover study; n=11 (Elite)	Simulated competition ballets;Old Rules	Lactate Data:Post-routine: 11.0 ± 1.9 mmol/L;Apnea Data:Duration: 64%.	Whole-body cryotherapy and active recovery accelerate lactate clearance and parasympathetic reactivation, mitigating the massive metabolic burden of apneic routines.	Simulated ballets in training
Shul’ga and Rudkovskaya (2013); Observational video analysis; n=32 (Elite)	Free routine (Solo);Old Rules	Apnea Data:Duration: 40%.	Elite free routines are characterized by extreme structural density, with a large proportion of time performed underwater to accommodate high-difficulty technical rotations.	Video analysis only; No physiological data
Tanner and Day (2017); Observational repeated-measures study; n=10 (Elite)	Intensified Training & Competition Period (No specific routine);Old Rules	Endocrine Data: Elevated salivary cortisol concentrations;	Elite artistic swimmers maintain mucosal immunity during intensified training but experience significant cortisol spikes driven primarily by psychological competition stressors.	Focus on salivary biomarkers; Limited physiological data
Bentley et al. (2022); Observational/Experimental study; n=6 (Elite)	Team Highlight routine;Old Rules	Lactate Data:Post-routine: 11.5 ± 1.09 mmol/L.	High movement frequencies combined with prolonged underwater time induce severe metabolic and respiratory acidosis (hypercapnia).	Small sample size (n=6); Simulated routine in training
Ramsay and Wolman (2001); Observational study; n=23 (Elite)	Treadmill test; Old Rules	VO2max: 47.2 ± 3.8 ml/kg/min.	Elite artistic swimmers maintain a protective level of body fat for buoyancy and thermoregulation, shielding them from amenorrhea despite intense training.	Lab test only; No AS routine
Yamamura et al. (2000); Observational/Experimental study; n=4 (Elite/Collegiate)	Team Technical, Team Free;Old Rules	Lactate Data:Post-routine:4.3 ± 1.1 mmol/L.	Lactate accumulation progressively increases throughout team routines, indicating a crucial shift to anaerobic glycolysis to fuel high-intensity lifts in the final phases.	Very small sample size (n=4); Simulated routines

Standard/FINA Guidelines: Performance was evaluated strictly following the official World Aquatics scoring standards, but comparing the effects of rule changes was not the primary focus. General: The study focused on the universal physiological characteristics or traditional paradigms inherent to artistic swimming, without explicitly distinguishing between the impacts of the pre-2023 old rules and the 2022–2025 new rules.

A synthesis of the literature indicates that study populations in artistic swimming research share distinct, sport-specific characteristics. Most included studies focused on female athletes. Competitive levels ranged from 7- to 14-year-old beginners (Badau, 2024) to elite national team members and Olympic medalists (Rodríguez-Zamora et al., 2014b). Several studies incorporated untrained females or athletes from other disciplines as control groups for cross-sectional physiological evaluations (Naranjo et al., 2006; Alentejano et al., 2010). Sample sizes are generally small (typically 4 to 34 participants), reflecting recruitment constraints among elite athletes and the technical challenges of underwater monitoring. Only a few cross-sectional investigations focusing on cardiac remodeling or morphological prediction achieved larger sample sizes of 60 to 92 participants (Coates et al., 2022b; Badau, 2024). Testing environments varied, encompassing in-competition monitoring (Elsherif, 2025), simulated routines in pools (Bante et al., 2007), laboratory-based incremental tests, and multi-week aquatic interventions (Fan et al., 2024). Interventional research has also explored performance optimization; for instance, wearing a jaw-repositioning intra-oral device (JID) during routines were found to elevate salivary cortisol levels without yielding significant improvements in blood lactate clearance or heart rate variability (HRV), acting primarily as an unadvisable additional stressor (Buscà et al., 2020).

The combination of high-intensity exertion and frequent apnea in artistic swimming necessitates specific measurement indices. Heart rate is a foundational metric, frequently used to capture the autonomic fluctuations between the apnea-induced diving reflex and the sympathetic excitation from surface movements (Figura et al., 1993; Solana-Tramunt et al., 2019). Blood lactate and peak oxygen uptake primarily quantify anaerobic metabolic stress and aerobic capacity. Rating of perceived exertion (RPE) and the proportion of facial immersion time are commonly assessed load indicators in field tests. Due to the challenges of underwater testing, limited studies have employed near-infrared spectroscopy (NIRS) for tissue oxygenation (Jones and Cooper, 2018) or echocardiography for left ventricular remodeling (Coates et al., 2022b).

Beyond acute cardiovascular and metabolic responses, the sport’s traditional aesthetic demands impose chronic physiological stress. Observational studies indicate that collegiate artistic swimmers frequently report low caloric intake and deficient Energy Availability (EA) (Costa et al., 2018). Combined with high training volumes and psychological stress, low EA alters resting metabolic rate (RMR) predictions. It also presents potential risks to bone mineral density (BMD), immune function, and overall health (Costa et al., 2018). Earlier research has also highlighted specific physiological adaptations to these demands. Elite artistic swimmers maintain an average maximal oxygen uptake (VO_2_max) of 47.2 ml/kg/min and rarely present with amenorrhea, unlike athletes in other aesthetic sports (Ramsay and Wolman, 2001). This relative protection against the Female Athlete Triad has been attributed to the need to maintain a baseline body fat percentage (approximately 23.0%) for buoyancy, alongside potential neuroendocrine adaptations to cold-water immersion (Ramsay and Wolman, 2001). These findings indicate a sport-specific balance among body composition, metabolic load, and endocrine health.

A notable gap in ecological validity remains across the literature: most existing data reflect historical regulatory frameworks. Under previous rules, judges favored routines with extended breath-holds and relatively slower movements. Consequently, earlier research predominantly focused on hypoxic risks and lactate accumulation associated with long, single apnea events (Davies et al., 1995; Alentejano et al., 2008). In contrast, recent studies explicitly conducted under the 2022–2025 scoring system remain limited (Fan et al., 2024; Badau, 2024). The new regulations emphasize choreographed standardization and assign higher difficulty values to underwater duration and technical execution. This has resulted in modern routines under the new scoring system containing denser lift sequences and complex leg combinations. Based on theoretical models projecting the impact of new rules, researchers recommend shifting choreography toward shorter, high-frequency apneic bouts with a 1:1 work-to-breathing ratio to prevent performance decline (Elsherif, 2025). These structural modifications alter the physiological and conditioning demands of the routines. The requirement for athletes to balance movement quality with extended underwater time underscores the need to re-evaluate physiological load data under the current scoring system.

### 3.2 Specific impacts of the new scoring system on acute physiological load and apnea characteristics

Artistic swimmers undergo dynamic acute physiological responses during routine execution.

#### 3.2.1 Heart rate dynamics

Heart rate (HR) in artistic swimming exhibits a fluctuating pattern characterized by pronounced bradycardia during apneic segments and rapid tachycardia during explosive surface movements (Figura et al., 1993). In elite athletes, peak HR typically averages 184.8 ± 6.6 bpm during technical and free duet routines (Rodríguez-Zamora et al., 2014a), whereas it reaches 191.3 ± 9.2 bpm in junior athletes and 192.4 ± 8.0 bpm in senior athletes across solo, duet, and team routines (Rodríguez-Zamora et al., 2012). Furthermore, mean peak heart rates have been recorded as high as 203.8 ± 5.0 bpm during high-intensity free duet routines (Iglesias et al., 2025).Conversely, mean minimum heart rate (HRmin) during facial immersion drops to 71.9 ± 16.6 bpm (Iglesias et al., 2025), with individual extremes historically recorded as low as 50.8 bpm (Rodríguez-Zamora et al., 2014a). Notably, trained swimmers exhibit more pronounced bradycardia during 20- to 25-second underwater bouts than untrained controls (Alentejano et al., 2012). In legacy contexts, these acute HR variations have been interpreted as reflecting the concurrent sympathetic activation and vagally mediated diving reflex inherent to the sport (Figura et al., 1993; Iglesias et al., 2025).

#### 3.2.2 Cardiac morphological adaptations

Chronic exposure to these acute hemodynamic fluctuations facilitates long-term cardiovascular remodeling. Echocardiographic data indicate increased left ventricular posterior wall thickness and left atrial enlargement in elite artistic swimmers (Coates et al., 2022b). Cross-sectional analyses further reveal that structural cardiovascular dimensions and aortic elastic properties in elite athletes significantly differ from those in varsity and junior counterparts (Baker et al., 2024), aligning with the physiological demands of specialized, long-term training.

#### 3.2.3 Apnea trends driven by 2022–2025 regulations

By reshaping routine structures, the 2022–2025 World Aquatics scoring system appears to have shifted choreographic demands. The revised regulations assign higher difficulty values to the accumulation and sequencing of complex movements (e.g., dense hybrid elements). Consequently, extended apnea is not a scoring criterion per se, but rather an unavoidable downstream consequence of executing these high-difficulty sequences (Elsherif, 2025; World Aquatics, 2022). Extrapolating from pre-2022 data, this increased density of prolonged apneic bouts suggests the hypothesis that athletes may now experience more sustained autonomic co-activation than under previous regulatory frameworks. To optimize performance amidst these demands, interventions such as jaw-repositioning intra-oral devices (JID) have been explored to facilitate underwater ventilatory mechanics without elevating the rating of perceived exertion (Buscà et al., 2020).

In conclusion, artistic swimming induces acute autonomic stress that drives chronic cardiovascular remodeling. By incentivizing extended apnea, the 2022–2025 regulations are hypothesized to alter the distribution of physiological load and potentially increase exposure to hypoxic environments (Elsherif, 2025). While existing data link traditional training to specific adaptations (Baker et al., 2024; Coates et al., 2022b), prospective longitudinal studies are essential to determine how the amplified acute loads under the current scoring system will influence long-term morphological remodeling.

### 3.3 Reported distributions of apnea characteristics and metabolic load indicators

Current literature provides a comprehensive overview of apnea duration, anaerobic glycolytic demands, and metabolic stress indicators within artistic swimming.

#### 3.3.1 Physiological monitoring of apnea duration and oxygenation levels

Apnea duration typically accounts for 45% to 64% of total routine duration across solo, duet, and free routines (Naranjo et al., 2006; Rodríguez-Zamora et al., 2018). While earlier observations recorded underwater proportions of approximately 45.11% (Naranjo et al., 2006), late traditional elite solo routines (Alentejano et al., 2008) and technical routines (Schaal et al., 2013) exhibit a significant shift, with apnea comprising up to 59–64% of the performance (Alentejano et al., 2008; Schaal et al., 2013).

Regarding respiratory parameters, early empirical research documented acute hypoxic stress, noting that alveolar oxygen partial pressure (p_A_O_2_) dropped to approximately 60 mmHg following apneic bouts during compulsory figures (Figura et al., 1993). Repeated-measures studies further indicate that chronic exposure to prolonged apnea leads to a compensatory decrease in baseline pulmonary diffusing capacity (García et al., 2021). Underwater near-infrared spectroscopy (NIRS) has revealed a rapid decline in tissue oxygen saturation in the vastus lateralis, while cerebral oxygenation remains relatively stable. This differential oxygenation suggests a robust blood flow redistribution mechanism to mitigate prolonged hypoxia (Jones and Cooper, 2018). Furthermore, interventions involving expiratory breath-holds (EBH) report arterial oxygen saturation (S_p_O_2_) falling to 88.4%, highlighting the severe hypoxemia inherent in high-intensity competitive scenarios (Guézennec et al., 2026).

#### 3.3.2 Blood lactate accumulation and anaerobic contributions

Metabolic load has evolved alongside choreographic complexity. Early team routines typically maintained post-performance blood lactate concentrations at 4.3 ± 1.1 mmol/L (Yamamura et al., 2000). In contrast, elite athletes exhibit blood lactate concentrations ranging from 5.93 ± 1.41 mmol/L following free duet routines (Iglesias et al., 2025) to 11.5 ± 1.09 mmol/L following team highlight and technical and free duet routines (Bentley et al., 2022). Values exceeding 11.0 mmol/L reflect anaerobic demands comparable to a 400-meter maximal all-out swim (Chatard et al., 1999; Schaal et al., 2013). Recent trials using extracellular buffering agents during simulated duet routines recorded baseline peak blood lactate at 8.4 ± 0.9 mmol/L, which increased to 9.3 ± 1.0 mmol/L when using extracellular buffering agents, suggesting that such interventions may support anaerobic propulsive force under extreme metabolic stress (Sprenger et al., 2025).

#### 3.3.3 Impact of the 2022–2025 scoring system on metabolic homeostasis

The 2022–2025 scoring system has incentivized shifts in choreographic structure and potential changes in metabolic distribution. By assigning higher difficulty values to complex hybrid sequences, the regulations categorize elements by underwater duration (e.g., TU3-level elements lasting ≥ 16 seconds) (Elsherif, 2025). However, within the judging framework, this extended apnea functions as a downstream consequence of accumulating dense leg combinations rather than a direct reward for breath-holding per se, ultimately compounding high-intensity technical exertion with extreme hypoxic stress. In a context where direct in-competition data under the new rules are limited, extrapolations from recent monitoring data suggest that these rule-driven changes may enhance the relative contribution of anaerobic glycolysis, a shift potentially correlated with intensified metabolite accumulation and potential declines in performance quality toward the end of routines (Elsherif, 2025; Iglesias et al., 2025).

In summary, the evolution of artistic swimming toward higher quantification standards has increased the relative time spent in apnea. Routines from the late traditional era are characterized by greater arterial hypoxia and a heightened reliance on anaerobic metabolism, as evidenced by elevated blood lactate levels. The 2022–2025 regulatory framework appears to exacerbate these physiological demands, potentially increasing the risk and severity of post-exercise hypoxemia.

### 3.4 Interactions between psychological load and the “Base Mark” mechanism

Emerging research emphasizes the intricate interplay between cognitive load, psychological stress, and physiological performance indicators in artistic swimming.

#### 3.4.1 Subjective perception and endocrine stress baselines

Elite athletes consistently report near-maximal fatigue following competitive routines, with ratings of perceived exertion (RPE, Borg CR-10) typically ranging between 7.5 and 7.9 (Rodríguez-Zamora et al., 2014a). This subjective strain is mirrored by objective endocrine responses; continuous monitoring of salivary biomarkers indicates that anticipatory psychological stress significantly potentiates post-competition salivary cortisol concentrations (Tanner and Day, 2017). Similar elevations in cortisol have been observed following specialized high-intensity training (Buscà et al., 2020), reflecting the combined impact of physical exertion and the high psychological demands of technical execution.

#### 3.4.2 Psychological stress and HRV suppression under the “Base Mark” framework

The “Base Mark” mechanism introduced in the 2022–2025 regulations has introduced a unique assessment environment that heightens cognitive demand. Maintaining technical precision in a high-stress, hypoxic, and dynamic underwater environment increases vigilance demands on the brain’s central executive network. Maintaining technical precision in a high-stress, hypoxic environment increases vigilance demands. Intense apneic episodes trigger strong parasympathetic activation (diving bradycardia), which heavily confounds traditional HRV assessments (Solana-Tramunt et al., 2019). Furthermore, this psychological and physical burden correlates with significant elevations in stress-induced endocrine markers, such as salivary cortisol (Tanner and Day, 2017).

#### 3.4.3 Inhibitory effects of cognitive fatigue on performance

The cumulative cognitive load under the revised regulations directly modulates athletic output. Experimental data demonstrate that athletes experiencing cognitive fatigue exhibit impaired executive function, manifested as prolonged reaction times and a significant reduction in the vertical height of explosive movements (e.g., surface jumps) (Ntomali et al., 2025). Interestingly, following cognitive fatigue interventions, researchers hypothesize that reduced glycolytic activation may occur, suggesting that under extreme psychological stress, the brain may implement a “self-protective” pacing strategy (Ntomali et al., 2025). This suggests that under extreme psychological stress, the brain may implement a “self-protective” pacing strategy, downregulating peripheral motor unit recruitment to manage the high uncertainty of the scoring system, rather than reaching a true peripheral metabolic limit.

Summary: The “Base Mark” penalty mechanism within the current scoring system appears to exacerbate athletes’ cognitive load. This interaction challenges homeostatic recovery through sustained cortisol elevation and suppressed autonomic reactivity, potentially creating a bottleneck for performance during high-stakes competition.

### 3.5 Physiological characteristics and apnea profiles across age groups

Existing literature underscores distinct heterogeneities in metabolic demands, cardiovascular adaptations, and apnea profiles between senior and junior/youth artistic swimmers.

#### 3.5.1 Differences in anaerobic metabolism and cardiovascular adaptation

Metabolic capacity and work output vary significantly across developmental stages. Longitudinal tracking of adolescent swimmers (mean age 11 years) demonstrated that absolute peak anaerobic power increased from 147.12 W to 210.17 W over one year of specialized training (Dzimbova and Markov, 2023). However, cross-sectional analyses reveal that peak blood lactate concentrations in Youth athletes (13–15 years) remain significantly lower than those in senior counterparts (>18 years) following maximal exertion, reflecting the developmental maturation of the glycolytic system (Bante et al., 2007; World Aquatics, 2023). Regarding cardiovascular remodeling, elite senior athletes exhibit profound resting bradycardia (57 ± 9 bpm) (Coates et al., 2022a), whereas junior female athletes present heart rates (72.70 ± 9.18 bpm) comparable to age-matched competitive swimmers, suggesting a lack of highly specific cardiac remodeling at younger ages (Rančić et al., 2024).

#### 3.5.2 Apneic responses and hypoxic risks under developmental constraints

Baseline apnea tolerance in younger cohorts (ages 7–14) is primarily limited by somatic maturity and biological age (Badau, 2024). Morphofunctional models identify “hypoxic tolerance” as a pivotal factor for early foundational success (Podrihalo et al., 2021). However, extreme apnea during vigorous exercise poses significant physiological risks; early field observations noted that elite athletes performing consecutive underwater bouts exhibited acute symptoms of severe hypoxia, including cyanosis and transient cognitive impairment (Davies et al., 1995). Epidemiological data further identify prolonged underwater breath-holding as a critical risk factor for shallow water blackout (SWB), a mechanism driven by blunted carbon dioxide buffering and a delayed ventilatory drive (Boyd et al., 2015).

#### 3.5.3 Regulatory evolution and age-specific safety policies

Under the 2022–2025 scoring system, routine components are strictly categorized into Elements (which include free hybrids, acrobatics, and technical required elements) and Transitions (World Aquatics, 2025a). The revised framework establishes unified quantitative standards for Time Underwater (TU) during hybrids, explicitly classifying them into TU1 (≤ 6 seconds), TU2 (7–15 seconds), and TU3 (≥ 16 seconds), incentivizing TU3-level elements (Elsherif, 2025; World Aquatics, 2025a). In response to the associated physiological risks, policy divergence has emerged. Notably, European Aquatics announced that, effective 2025, hybrid elements exceeding 25 seconds in Junior category will incur a mandatory penalty (score reduced to the Base Mark) to safeguard adolescent athletes (SwimSwam, 2025). Current regulations strategically impose this limit on the Junior developmental cohort.

Summary: Physiological adaptation in artistic swimming is highly age-dependent. Junior athletes exhibit immature anaerobic tolerance and cardiovascular remodeling, with apnea capacity constrained by developmental stages. The increased prevalence of prolonged underwater choreography under current regulations may exacerbate the risk of acute hypoxemia, such as shallow water blackout, in younger populations. Current restrictive policies for youth competitions represent a pragmatic effort by governing bodies to balance technical difficulty with the physiological safety thresholds inherent to growth and development.

## 4 Discussion

### 4.1 Summary of main findings

This scoping review systematically maps the physiological evidence landscape of artistic swimming under the 2022–2025 World Aquatics scoring system. A direct comparison of the key physiological and psychological indicators between the traditional and the new regulatory eras is summarized in **Table 3**. Based on theoretical extrapolation from legacy data, emerging trends indicate that by incentivizing high-difficulty hybrid elements (TU3)—which inherently require extended underwater execution through higher difficulty weighting, the revised regulations likely amplify the physiological and metabolic demands of the sport. A central theme identified in the literature is the characteristic “autonomic conflict”—a physiological antagonism between sympathetic activation driven by high-intensity surface exertion and the vagally mediated diving reflex triggered by apnea.

**Table 3:** Comparison of physiological and psychological indicators between traditional (pre-2022) and new (2022-2025) rules era.

Indicator (physiological/psychological metric)	Traditional era (baseline/old rules data)	New rules era (2022–2025 data)	Scientific evidence (author, year)
Apnea Time Ratio (%)	**~40% - 64%** ②Early traditional routines recorded ~40%–45.11% underwater time. ②In the late old-rules era, elite routine apnea time progressively increased, reaching 59%–64%. ②And In the late traditional era (Tokyo 2020 Olympics), the average underwater time in elite free routines was directly recorded at 57.8%	③**NR (Not Reported)** Under the 2022–2025 regulations (which utilize underwater duration categories like TU3 to structure difficulty weights for complex movements), there are no direct empirical measurements yet. However, theoretical projections and strategic models recommend limiting underwater time to ~50% (a 1:1 ratio) to prevent hypoxia-induced penalties in execution scores	Old Rules: Naranjo et al. (2006); Shul’ga and Rudkovskaya (2013); Alentejano et al. (2008); Rodríguez-Zamora et al. (2018). Elsherif (2025). New Rules: NR(Empirical); Elsherif (2025) (Theoretical).
Peak Blood Lactate (mmol/L)	**3.4 - 11.5 mmol/L** ②Early routines elicited moderate peak lactate (~4.3 - 4.8 mmol/L). ②In later high-difficulty choreographies, post-exercise peak blood lactate soared to~5.93–11.5 mmol/L (including recent simulations averaging 8.4 ± 0.9 mmol/L).	③**NR (Not Reported)** Current empirical studies evaluating routines under the 2022–2025 regulations have not reported direct post-exercise peak blood lactate measurements	Old Rules: Figura et al. (1993); Yamamura et al. (2000)); Bentley et al. (2022); Sprenger et al. (2025) New Rules: NR.
Cardiovascular Stress (Heart Rate Dynamics)	**HRmin 56.5 bpm to HRpeak 203.8 bpm** ②Routines induced significant autonomic conflict, alternating between severe apneic bradycardia (dropping to ~56.5–71.9 bpm) and exercise-induced tachycardia (peaking at ~180.4–203.8 bpm).	③**NR (Not Reported)** Specific in-competition heart rate dynamics and extreme HR values under the 2022–2025 scoring system have not been quantitatively reported in the included literature.	Old Rules: Rodríguez-Zamora et al. (2012); Rodríguez-Zamora et al. (2014b); Iglesias et al. (2025). New Rules: NR.
Psychological & Cognitive Load	**RPE 7.5–7.9/Cortisol Spikes** ②Subjective fatigue was rated as “extremely hard” (RPE 7.5–7.9).②Anticipatory psychological stress significantly elevated salivary cortisol concentrations during competition.	③**NR (Not Reported)** Currently, there are no direct empirical measurements quantifying the specific cognitive load induced by the “Base Mark” penalty mechanism under the 2022–2025 regulations. ②However, simulated tests inducing mental fatigue (via 30-min Stroop tasks) demonstrate that impaired central executive function leads to reduced technical performance scores and decreased boost height (from 72 ± 5 cm to 70 ± 5 cm).	Old Rules: Rodríguez-Zamora et al. (2014a); Tanner and Day (2017). New Rules/FINA Guidelines: Ntomali et al. (2025).
Hypoxic Stress (SpO2/pAO2)	**pAO2 3.67 KPa/SpO2 ~88.4%** ②During extreme underwater sequences in traditional free routines, alveolar PO2 dropped to 3.67–5.07 KPa.②Additionally, specific off-routine intensive breath-hold sprint training interventions induced arterial desaturation, reducing SpO2 to ~88.4%.	③**NR (Not Reported)** Direct blood gas parameters (pAO2) or arterial oxygen saturation (SpO2) during routine execution under the 2022–2025 rules have not been reported.	Old Rules/General: Figura et al. (1993); Davies et al. (1995); Guézennec et al. (2026). New Rules: NR.
Adolescent Safety Risk	**Developmental Constraints & Hypoxia Symptoms** ②Youth athletes present lower anaerobic maturity (lower peak lactate). ②Elite apneas historically caused cyanosis and mild confusion (SWB risk).	③**NR (Not Reported)** Direct empirical data measuring the physiological responses of youth athletes under the 2022–2025 scoring system have not been reported. ③Based on theoretical physiological risks incentivized by the new system, European Aquatics (EA) mandated a strict 25-second underwater apnea penalty limit for the Junior category effective 2025.	Old Rules: Bante et al. (2007); Davies et al. (1995). New Rules: SwimSwam (2025).

Evidence Strength Legend: ① Direct Empirical Evidence: Actual measurement data obtained under the 2022–2025 regulations (Red text). ② Indirect Evidence: Results derived from simulation tests or historical data based on previous rules (Yellow text). ③ Theoretical Projection: No direct measurement data available (NR); hypotheses formulated based on established physiological principles and theoretical extrapolation (Blue text).

Bold values denote key summary values; bold ‘NR’ denotes no direct empirical data under the 2022–2025 regulations.

While theoretical frameworks suggest this autonomic antagonism may harbor latent safety risks, and empirical studies report substantial lactate accumulation and arterial hypoxia, direct evidence linking the new regulations to long-term cardiac remodeling or acute clinical events in elite cohorts remains sparse (Elsherif, 2025; Williams et al., 2024).

Furthermore, regarding the “Base Mark” penalty mechanism, recent investigations suggest an intricate psycho-physiological interplay. Preliminary evidence indicates that maintaining technical precision under severe hypoxic conditions imposes an extraordinary cognitive load on the central executive network. This psychological strain appears to correlate with diminished anaerobic output and compromised underwater explosive power (Ntomali et al., 2025), suggesting that the “Base Mark” environment may act as a catalyst for cognitive fatigue-induced performance decline.

Finally, this review highlights critical developmental constraints in adolescent populations, particularly regarding CO_2_ buffering and anaerobic metabolic capacity. Integrating evidence from emergency medicine—where prolonged apnea is a documented risk factor for shallow water blackout—it is evident that the regulatory push for extended underwater time may challenge the physiological safety thresholds of younger athletes (Boyd et al., 2015). Given that current data largely derive from training environments under legacy frameworks, there is an urgent need for ecologically valid, prospective studies to accurately quantify the impact of the 2022–2025 scoring system on athlete health and safety across the developmental spectrum.

### 4.2 The physiological cost of the new scoring system: autonomic conflict and cardiac remodeling

Before delving into specific physiological responses, it is crucial to acknowledge that owing to the scarcity of in-competition monitoring under the current regulations, the anticipated impacts of the 2022–2025 scoring system discussed herein represent theoretical extrapolations grounded in well-established historical baselines.

Section 3.2 delineates the profound heart rate fluctuations experienced by artistic swimmers, with minimums dropping to ~56.5 bpm and peaks reaching ~203.8 bpm. These hemodynamic oscillations epitomize the “autonomic conflict” inherent to the sport: high-intensity surface movements trigger potent sympathetic activation to meet muscular perfusion demands, while subsequent facial immersion and prolonged apnea elicit a vagally mediated diving reflex, inducing rapid bradycardia and peripheral vasoconstriction (Shattock and Tipton, 2012; Williams et al., 2024). However, applying the “autonomic conflict” construct directly to artistic swimming requires careful contextualization. The original framework was developed to explain sudden death during unexpected, cold-water immersion under severe thermal stress (Shattock and Tipton, 2012). In stark contrast, competitive artistic swimming is performed voluntarily by highly adapted athletes in controlled, thermoneutral environments, with official pool temperatures strictly regulated to a minimum of 27 °C (World Aquatics, 2025b). Furthermore, trained athletes exhibit functional adaptations to hypoxia and engage in repeated re-oxygenation through intermittent surface breathing during routines, mitigating continuous asphyxia (Alentejano et al., 2008). Therefore, using the original cold-water conflict model directly as a mechanistic substrate for predicted arrhythmogenesis in artistic swimming represents a non-trivial extrapolation.

The 2022–2025 scoring system assigns higher difficulty weights to TU3-level hybrid elements lasting ≥ 16 seconds (Elsherif, 2025). This regulatory shift incentivizes routines characterized by more frequent and sustained underwater exertion, potentially increasing the prevalence of autonomic co-activation. In related physiological research, the robust concurrent activation of the sympathetic nervous system and the diving reflex has been proposed to challenge myocardial electrical stability and increase ventricular repolarization heterogeneity (Shattock and Tipton, 2012). However, direct empirical evidence quantifying the acute cardiac safety implications of these rule-driven changes in artistic swimmers remains sparse.

Regarding long-term morphological adaptations, the increased left ventricular posterior wall thickness and left atrial enlargement identified in Section 3.2 (Coates et al., 2022b) reflect specific remodeling in response to the dual stressors of volume overload and apnea-induced pressure changes. Although the revised regulations may amplify exposure to these hemodynamic stimuli, current interpretations classify these shifts as exercise-induced physiological adaptations rather than pathological markers. Given the significant heterogeneity in existing data—most of which predate the current regulatory framework—there is presently no direct evidence linking the 2022–2025 scoring system to an increased risk of pathological remodeling or structural heart disease in elite cohorts (Coates et al., 2022b).

### 4.3 Metabolic load and hypoxia: breath-holding characteristics and potential implications

The indices mapped in Section 3.3 regarding high-intensity anaerobic glycolytic demands and hypoxia underscore a unique physiological challenge in artistic swimming. The observation by Jones and Cooper (2018) of stable prefrontal oxygenation despite a decline in peripheral muscle tissue saturation (TSI%) reflects a localized blood flow redistribution mechanism. In the context of diving physiology, this phenomenon—characterized by robust peripheral vasoconstriction—prioritizes cerebral oxygen supply at the expense of limb perfusion (Bouten et al., 2021; Lindholm and Lundgren, 2009). Such mechanistic patterns suggest that lower-limb musculature is compelled to enhance its reliance on non-mitochondrial pathways within a relatively ischemic environment.

Data from Section 3.3 further demonstrate that peak post-routine blood lactate concentrations in elite athletes under pre-2022 regulations reach up to 11.5 mmol/L, accompanied by significant reductions in arterial oxygen saturation. Following established bioenergetic models, these elevated lactate levels serve as biomarkers of rapid ATP hydrolysis and the associated release of protons (H^+^), rather than being the direct cause of metabolic acidosis (Robergs et al., 2004). In fundamental metabolic research, the accumulation of free H^+^ and inorganic phosphate (P_i_) has been shown to interfere with excitation-contraction coupling, ultimately precipitating neuromuscular fatigue and performance decrements. While these biochemical mechanisms derive from general exercise physiology, they provide a robust theoretical framework for understanding the performance decline associated with the superimposed stressors of peripheral ischemia and apnea in this sport.

While the 2022–2025 scoring system utilizes underwater duration categories (such as TU3) to structure difficulty weights (Elsherif, 2025), extended breath-holding remains a downstream consequence of completing complex movement sequences rather than a primary scoring criterion. This regulatory architecture objectively incentivizes the accumulation of movement difficulty, thereby systematically exposing athletes to high-frequency, prolonged apneic conditions. This regulatory shift potentially facilitates the cumulative effects of metabolic by-products (H^+^ and P_i)_, imposing rigorous challenges on athletes’ anaerobic tolerance. However, it must be emphasized that current extreme metabolic and hypoxic values derive predominantly from legacy regulatory frameworks. Although trends suggest an amplified metabolic burden under the new rules, direct research with high ecological validity in authentic competitive settings remains sparse. Consequently, the precise magnitude of the “metabolic crisis” induced by the new scoring system in elite athletes remains to be established through future prospective studies.

### 4.4 Psycho-physiological interactions: the “Base Mark” penalty and cognitive fatigue

Section 3.4 delineates the psychophysiological strain experienced in artistic swimming, characterized by elevated cortisol concentrations and increased ratings of perceived exertion (RPE) under competitive stress (Rodríguez-Zamora et al., 2014a; Tanner and Day, 2017). Interdisciplinary frameworks from sports science provide a robust theoretical lens to interpret these psycho-physiological interactions. According to the psychobiological model of endurance performance, motor output is not solely constrained by peripheral physiological fatigue but is fundamentally modulated by the brain’s perception of effort (Marcora et al., 2009; Pageaux, 2014). Specifically, Pageaux’s (2014) effort-based decision-making theory postulates that elevated cognitive load may amplify corollary discharge, leading to a premature escalation in RPE. This mechanism suggests that athletes may subconsciously adopt a “self-protective” pacing strategy to mitigate perceived strain. This perspective elucidates the paradoxical observations identified in Section 3.4: following cognitive fatigue interventions, athletes exhibit suppressed post-simulation blood lactate concentrations alongside decrements in explosive jump height (Ntomali et al., 2025). Such findings imply that under high cognitive load, the central nervous system may downregulate peripheral motor unit recruitment—particularly of high-threshold fast-twitch fibers—rather than reflecting a genuine physiological shift in anaerobic capacity.

Furthermore, psycho-physiological interactions substantially modulate post-exercise recovery. Drawing upon the neurovisceral integration model, sustained cognitive anxiety and anticipatory stress are proposed to impair prefrontal inhibitory control over the amygdala, maintaining the sympathetic nervous system in a state of hyper-arousal (Thayer et al., 2012). These neurophysiological inferences offer a framework for understanding how stress-induced cortisol elevations impede the restoration of vagal tone, resulting in the prolonged suppression of heart rate variability (HRV) observed following routines (Solana-Tramunt et al., 2019). However, it must be noted that these mechanistic explanations currently rely on foundational psychophysiological models. Empirical research directly linking the “Base Mark” penalty mechanism to cognitive fatigue in authentic competitive environments remains sparse. Future investigations must prioritize evidence with high ecological validity to ascertain the precise impact of rule-driven psychological load on the performance and systemic recovery of artistic swimmers.

### 4.5 Age-group differences and potential safety implications in adolescent athletes

Section 3.5 delineates the distinct physiological adaptation profiles differentiating adolescent from elite senior athletes. During high-intensity routines, adolescent swimmers (aged 13–15 years) typically exhibit lower peak blood lactate concentrations than their senior counterparts (Bante et al., 2007). In developmental physiology, this disparity is interpreted as a manifestation of the maturational status of the anaerobic glycolytic system and chemical buffering capacity (Beneke et al., 2007; Ratel et al., 2006).

Foundational evidence suggests that a lower bicarbonate buffering capacity implies adolescents possess a diminished ability to maintain pH homeostasis when confronted with the synergistic stressors of respiratory acidosis (induced by apnea) and metabolic acidosis (triggered by exertion) (Beneke et al., 2007). These characteristics underscore a specific physiological vulnerability of younger cohorts under extreme hypoxic loads.

The 2022–2025 World Aquatics (2022) scoring system incentivizes the inclusion of highly complex hybrid and acrobatic elements, which inherently demand prolonged apnea for successful execution. Given that apnea tolerance in adolescents is heavily constrained by biological maturity (Badau, 2024), this regulatory framework potentially escalates physiological load exposure. In public health and emergency medicine, hazardous breath-holding is a well-documented risk factor for shallow water blackout (SWB)—a phenomenon where the partial pressure of oxygen (P_O_2) drops below the threshold for consciousness before the accumulation of CO_2_ can trigger a sufficient urge to breathe (Boyd et al., 2015).Early reports have documented emergency cases of hypoxic syncope among adolescent artistic swimmers during prolonged training (Quan et al., 2010), suggesting that the pursuit of extended underwater sequences during specific developmental stages carries latent safety risks.

In response to this trend, European Aquatics (EA) mandated a 25-second underwater safety limit specifically for the Junior category effective 2025 (SwimSwam, 2025). Although this specific regulation targets the Junior category, the physiological vulnerabilities inherent to early developmental stages suggest that younger athletes (e.g., Youth and U12) may face equivalent or heightened risks, underscoring the necessity of evaluating downward extensions of such protective policies. This policy divergence reflects a proactive shift by international governing bodies toward mitigating age-specific physiological risks. However, while mechanistic inferences and historical cases suggest potential hazards, direct empirical evidence linking the 2022–2025 regulations to an increased incidence of SWB in contemporary athletes is currently absent. Most extreme hypoxic data derive from legacy training frameworks. Future research urgently requires longitudinal epidemiological data with high ecological validity to accurately quantify the impact of the current scoring system on the physiological safety of athletes across the developmental spectrum.

### 4.6 Limitations and gaps in the current literature

While this review provides a systematic mapping of the physiological landscape in artistic swimming, several critical gaps in the existing evidence base must be acknowledged. A fundamental limitation is that most of the 36 included empirical studies were conducted in competitive environments predating the 2022–2025 regulatory reforms (Viana et al., 2019). Under the theory of “representative design” in sports science, experimental task constraints must align with actual environmental constraints to ensure the ecological validity and transferability of findings (Pinder et al., 2011).

Since the current scoring system is hypothesized to alter choreographic density and metabolic demands, considerable uncertainty remains regarding the extent to which “pre-revision” baseline data accurately reflect the acute physiological stress now experienced by elite athletes. From an ecological dynamics perspective, athletic performance is an emergent property of the continuous interplay between the athlete and specific environmental constraints (Woods et al., 2020). Consequently, the current lack of high-resolution, real-time physiological monitoring during authentic competitions under the new regulations represents a significant void in ecologically valid research.

Furthermore, the methodological scope of this scoping review is subject to specific constraints. The search strategy was restricted to major English-language academic databases (e.g., PubMed, Web of Science). Given the global competitive landscape of artistic swimming, it is plausible that nations with long-standing dominance in the sport (e.g., Russia) have conducted localized medical and physiological monitoring. Due to language barriers, such specialized data published in non-English journals were not integrated into this qualitative synthesis. This potential language and publication bias may result in the omission of critical physiological adaptation profiles from some of the world’s highest-level cohorts.

### 4.7 Practical implications and future directions

Synthesizing the evidence mapped in this review alongside anticipated physiological trends under the 2022–2025 scoring system, this study delineates critical implications for training monitoring, medical support, future research, and regulatory optimization.

#### 4.7.1 Implications for training choreography and load monitoring

Coaching teams are encouraged to balance the pursuit of quantified difficulty with the physiological constraints of metabolic clearance. Given that prolonged apnea coupled with high-intensity exertion induces substantial blood lactate accumulation (Bentley et al., 2022; Iglesias et al., 2025), choreography should strategically integrate respiratory recovery intervals between dense hybrid sequences (e.g., TU_3_-level elements). Furthermore, practitioners should adopt heart rate variability (HRV) and ratings of perceived exertion (RPE) as indispensable daily monitoring metrics. These tools facilitate the identification of psychological strain and autonomic fluctuations associated with the “Base Mark” penalty mechanism, thereby optimizing the evaluation of an athlete’s holistic recovery status (Solana-Tramunt et al., 2019).

#### 4.7.2 Considerations for medical support during competitions

Given the potential health risks inherent to extreme hypoxic environments (Quan et al., 2010), safety protocols at major events must align with the specific choreographic and physiological profiles of artistic swimming. It is recommended that medical personnel specifically trained in aquatic hypoxia recognition be stationed at both competitive and high-intensity training venues. Establishing comprehensive, equipment-supported poolside protocols—including portable oxygen therapy, automated external defibrillators (AEDs), and pulse oximeters—is essential to expedite interventions during acute hypoxic events and mitigate catastrophic safety risks (Boyd et al., 2015; Quan et al., 2010).

#### 4.7.3 Perspectives on future research and regulatory optimization

The evolution of competitive regulations necessitates a data-driven balance between technical advancement and physiological tolerance. Regarding the 2022–2025 World Aquatics framework, future research urgently requires studies with high ecological validity to quantify the precise metabolic cost of high-frequency underwater elements. For developing athletes, the 25-second underwater limit mandated for the Junior category by European Aquatics (EA) provides a commendable policy template (SwimSwam, 2025). Future international regulatory revisions should evaluate the necessity of duration-based restrictions grounded in age-specific biological evidence. Moreover, quantitative assessments of the “Base Mark” mechanism’s impact on cognitive load are required to develop evidence-based models that safeguard athlete well-being without compromising technical excellence, as emphasized in recent clinical perspectives (Williams et al., 2024).

## 5 Conclusions

This scoping review systematically maps and synthesizes existing evidence regarding the physiological load and apnea characteristics of artistic swimming. Current findings underscore that physiological demands in this sport are defined by a complex interplay between high-intensity dynamic exertion and apnea-induced responses, including autonomic antagonism, substantial anaerobic metabolic stress, and specific psycho-physiological interactions. Based on theoretical extrapolation from legacy data, the 2022–2025 scoring system—specifically the increased difficulty weights for prolonged underwater hybrid elements and the “Base Mark” penalty mechanism—is hypothesized to escalate athletes’ exposure to cardiovascular and metabolic loads, presenting unique safety considerations for physiologically developing adolescent populations. However, because the preponderance of current empirical data derives from studies predating the 2022 regulatory reforms, a substantial evidence gap currently exists. Direct empirical inferences regarding the acute and long-term impacts of these revisions remain constrained. Consequently, definitive conclusions concerning the precise clinical risks of the new regulations cannot yet be established. Future longitudinal studies, executed under authentic competitive conditions with high ecological validity, are urgently required to accurately quantify the impact of current regulations on the physiological safety of elite athletes across the developmental spectrum.

## Data Availability

The original contributions presented in the study are included in the article/**Supplementary Material**. Further inquiries can be directed to the corresponding author.
