# Supplementary material for: Physiological load and breath-holding in artistic swimming: a scoping review establishing historical baselines and evidence gaps in the context of the 2022–2025 rule changes
Source: Front Physiol. 2026 Jun 18;17:1855762. doi: 10.3389/fphys.2026.1855762 (PMC13322880; doi:10.3389/fphys.2026.1855762)
Supplement: Supplementary file 1 [file Table1.docx]

Database 1: PubMed

Date of Search: January 12, 2026

Search String:("artistic swimming"[Title/Abstract] OR "synchronized swimming"[Title/Abstract] OR "synchronised swimming"[Title/Abstract] OR synchro[Title/Abstract]) AND (physiological[Title/Abstract] OR "heart rate"[Title/Abstract] OR "oxygen uptake"[Title/Abstract] OR VO2[Title/Abstract] OR "blood lactate"[Title/Abstract] OR "oxygen saturation"[Title/Abstract] OR SpO2[Title/Abstract] OR "breath-hold*"[Title/Abstract] OR "breath holding"[Title/Abstract] OR apnea[Title/Abstract] OR apnoea[Title/Abstract] OR hypoxia[Title/Abstract])

Filters Applied: Language = English

Records Identified: 32

Database 2: Web of Science (Core Collection)

Date of Search: January 12, 2026

Search String: TS=("artistic swimming" OR "synchronized swimming" OR "synchronised swimming" OR synchro) AND TS=(physiological OR "heart rate" OR "oxygen uptake" OR VO2 OR "blood lactate" OR "oxygen saturation" OR SpO2 OR "breath-hold*" OR "breath holding" OR apnea OR apnoea OR hypoxia)

Filters Applied: Language = English; Document Type = Article

Records Identified: 59

Database 3: Scopus

Date of Search: January 12, 2026

Search String: TITLE-ABS-KEY("artistic swimming" OR "synchronized swimming" OR "synchronised swimming" OR synchro) AND TITLE-ABS-KEY(physiological OR "heart rate" OR "oxygen uptake" OR VO2 OR "blood lactate" OR "oxygen saturation" OR SpO2 OR "breath-hold*" OR "breath holding" OR apnea OR apnoea OR hypoxia)

Filters Applied: Language = English; Document Type = Article

Records Identified: 36

Database 4: Google Scholar (Supplementary Search)

Date of Search: January 19, 2026

Search String: ("artistic swimming" OR "synchronized swimming" OR "synchro") AND (physiology OR "heart rate" OR "blood lactate" OR VO2 OR apnea OR "breath-hold" OR "diving reflex" OR bradycardia)

Filters/Stopping Criterion: Language = English; Date Range = 2021-2026；Sorted by relevance

Records Identified: 76

Google Scholar Stopping Criterion: Screened the first 10 pages sorted by relevance. Following this manual screening, exactly 76 records that met the predefined date, language, and initial relevance criteria were exported for subsequent deduplication.

Deduplication Software: Duplicates were identified and removed using Zotero.

Inter-rater Agreement Metric: The initial independent screening of the 137 deduplicated records yielded a raw agreement rate of approximately 95%. In accordance with our protocol, all initial discrepancies regarding study inclusion were fully resolved via discussion or by consulting a third reviewer to achieve 100% final consensus.
